# Wandering along the epigenetic timeline

**DOI:** 10.1186/s13148-020-00893-7

**Published:** 2020-07-02

**Authors:** Clémence Topart, Emilie Werner, Paola B. Arimondo

**Affiliations:** 1 Department of Chemistry, Ecole Normale Supérieure, 24 rue Lhomond, 75005 Paris, France; 2 PSL Research University, 60 Rue Mazarine, 75006 Paris, France; 3 EpiCBio, Epigenetic Chemical Biology, Department Structural Biology and Chemistry, Institut Pasteur, CNRS UMR n°3523, 28 rue du Dr Roux, 75015 Paris, France

**Keywords:** Epigenetic clocks, Ageing, Environmental factors, Age-related pathologies, Reprogramming

## Abstract

**Background:**

Increasing life expectancy but also healthspan seems inaccessible as of yet but it may become a reality in the foreseeable future. To extend lifespan, it is essential to unveil molecular mechanisms involved in ageing. As for healthspan, a better understanding of the mechanisms involved in age-related pathologies is crucial.

**Main body:**

We focus on the epigenetic side of ageing as ageing is traced by specific epigenetic patterns and can be measured by epigenetic clocks. We discuss to what extent exposure to environmental factor, such as alcohol use, unhealthy diet, tobacco and stress, promotes age-related conditions. We focused on inflammation, cancer and Alzheimer’s disease. Finally, we discuss strategies to reverse time based on epigenetic reprogramming.

**Conclusions:**

Reversibility of the epigenetic marks makes them promising targets for rejuvenation. For this purpose, a better understanding of the epigenetic mechanisms underlying ageing is essential. Epigenetic clocks were successfully designed to monitor these mechanisms and the influence of environmental factors. Further studies on age-related diseases should be conducted to determine their epigenetic signature, but also to pinpoint the defect in the epigenetic machinery and thereby identify potential therapeutic targets. As for rejuvenation, epigenetic reprogramming is still at an early stage.

**Graphical abstract:**

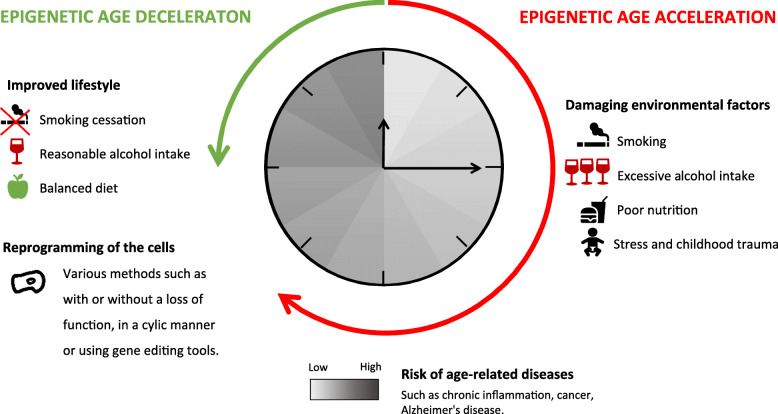

## Background

In the early 1940s, the British embryologist Conrad Waddington brought to light the fact that gene-environment interactions impact on development and embryology and called this phenomenon epigenetics [1]. This major breakthrough marks the beginning of uncountable studies of the epigenetic mechanisms that flourished in particular with the sequencing of the human genome in 2001. Thereafter, the definition of epigenetics became clearer on the molecular level. Several definitions have been discussed [2]. The most common describes epigenetics as “the study of heritable phenotypes that do not alter the DNA sequence” [3], and an operational definition is “Epigenetics simply brings to concepts what is beyond genes” [4]. In short, epigenetics describes all reversible and heritable processes that regulate gene expression without altering the genetic sequence. It has been shown that DNA and histones can undergo chemical modifications such as methylation and acetylation—for histones only—that will guide the winding of DNA around histones [5] and determine chromatin compaction. These chemical modifications are commonly called epigenetic “marks”. Interactions between DNA and histones thereupon lead either to a euchromatin conformation where the gene is accessible and therefore activated or to a heterochromatin conformation where the gene is inaccessible and thus repressed [6]. In addition to DNA and histone modifications, other machineries are involved in the epigenetic regulation, as nucleosome positioning [7] and non-coding RNAs [8]. Here, we have made the choice to focus on DNA and histone modifications that are the most studied in relation to ageing, even if it is important not to forget that all the epigenetic machineries are interlinked and influence each other [9, 10]. For example, DNA methylation deregulation induces changes in the expression of miRNA/lncRNA participating to ageing and age-related diseases [11–13].

The epigenetic proteins involved in DNA and histone modifications are the *writers*, *erasers* and *readers*. *Writers*’ proteins catalyse the transfer of the chemical modification on the DNA or histone, the *erasers*’ proteins remove it, and *readers*’ proteins interpret the chemical modification and trigger signalling pathways [14].

Ageing, a process that can be defined as the deterioration of physiological functions through time under normal environmental conditions [15] and as the weakening of the ability to adapt to metabolic stress [16], is closely linked to epigenetics. Epigenetic marks, in particular DNA methylation patterns, at specific genomic regions have been associated with ageing [17]. It is therefore conceivable to date and predict the lifespan of an individual by using specific epigenetic biomarkers.

Alteration of these patterns by environmental factors such as stress or alcohol use promotes age-related diseases [18]. A greater understanding of the molecular mechanisms involved in age-related diseases is essential to identify druggable targets but also to allow early diagnostic of the disease. Since epigenetic marks are reversible, treatments targeting the responsible epigenetic actors can be considered to cure age-related pathologies or even to reprogram cells [19–21].

In this perspective, we first discuss how environmental factors can induce epigenetic changes causing clock acceleration and thereby promoting age-related diseases. We also review the different epigenetic clocks based on DNA methylation, as they are key tools to study ageing. Finally, we provide examples of how to epigenetically reverse the time and thereby increase healthspan. Overall, this perspective aims at providing a broad overview on the causes and consequences of epigenetic age modulation based on three hypotheses. (1) Epigenetic age is more representative and relevant than chronological age to study ageing because the dynamic epigenetic modifications integrate the impact of the environment (natural, social, tissue-specific) and consequently induce a modification of the phenotype. As such, (2) the impact of environmental factors on ageing and age-related diseases is registered by the epigenetic modifications that can be used as “clocks”. (3) Since epigenetic modifications are chemically reversible, they can be targeted to reverse time and develop rejuvenation strategies.

## Epigenetic dating

The epigenome, describing all the epigenetic marks on DNA and histones, is not entirely maintained in somatic cells over ageing [22, 23]. This epigenetic drift is responsible for alteration of the number and type of proteins synthesized by the cell [23]. DNA methylation occurs at the cytosine’s C5 position on deoxycytidine-phosphate-deoxyguanosine (CpG) dinucleotides. The reaction is catalysed by DNA methyltransferases (DNMTs) and requires the *S*-adenosyl-l-methionine (SAM) cofactor as methyl donor [24]. Epigenetic drift translates into changes of DNA methylation patterns, where CpG islands on specific gene promoters are often hypermethylated, while a global hypomethylation is observed [25, 26]. As changes in the methylome result from random errors and environment influence, they differ from one individual to another [27].

**Fig. 1:**
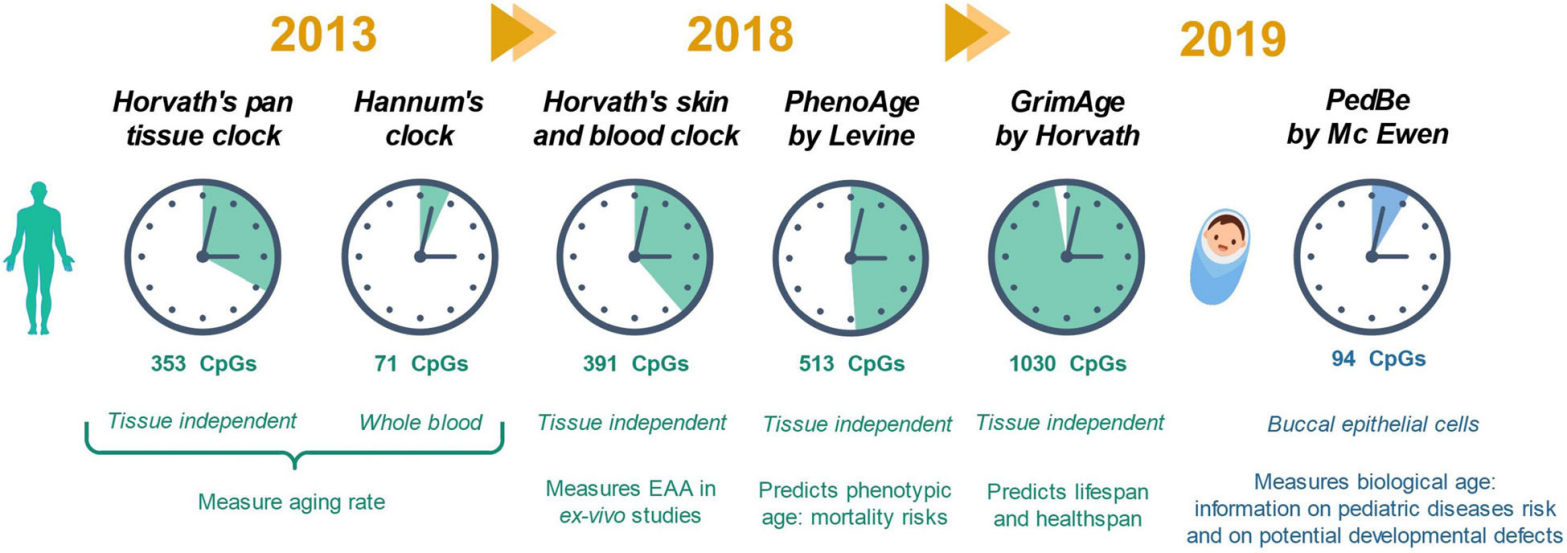
Epigenetic clocks discussed in this review. EAA: epigenetic age acceleration

Age can be predicted by performing a biological test based on DNA methylation levels, referred to as an epigenetic clock [28, 29]. Biological age should be distinguished from chronological age. While chronological age refers to the time elapsed since the birth of the individual, biological age is linked to the decline of biological functions. The biological and chronological ages of an individual with a healthy lifestyle should match. However, exposition to environmental factors such as stress and alcohol use increases biological age [30]. Epigenetic clocks measure epigenetic ageing and can provide information on both chronological and biological age depending on the studied CpG domains [31]. The epigenetic clocks discussed in this review are represented in Fig. 1.

The two first epigenetic clocks predicting chronological age were both reported in 2013 by Horvath et al. [28, 29] and Hannum et al. [32]. Hannum’s clock was developed on 656 people aged from 19 to 101 years old using whole blood samples. The model requires input of gender and body mass index. Methylation was measured using Illumina Infinium HumanMethylation 450 BeadChip assay, a high density DNA methylation array with single CpG site resolution [33]. The test was devised on a group of 482 people. First tests were performed taking approx. 485,000 CpG markers into account. It was then fine-tuned to a set of 71 markers showing strong methylation-age relationship. Interestingly, these markers are located near genes involved in age-related diseases. The model was then validated on a second group of 174 people and showed a correlation of 0.91 between chronological age and predicted age with an error of 4.9 years. However, the test is tissue specific, and its use on a tissue other than blood needs further calibration. Therefore, the clock does not allow to compare ageing in different tissues.

In contrast to Hannum’s clock, Horvath’s pan-tissue clock is tissue and cell type independent [28, 29]. It is an interesting tool to compare ageing in different organs and identify potential deficiencies. The test was calibrated using 21,369 CpG markers with the same methylation measure technique as Hannum [32, 33]. Horvath’s clock is based on 353 markers—among them, 160 CpG markers are negatively correlated with age—and offers greater precision than Hannum’s clock with an age correlation of 0.96 and an error of 3.6 years. Recent studies using Horvath’s and Hannum’s clocks to predict disease and mortality risk have been thoroughly reviewed by Fransquet et al. [34].

Horvath later developed another clock, the skin and blood clock [35] (Fig. 1), more accurate in terms of age correlation and median error and based on 391 CpGs. This clock aims at predicting age within the framework of ex vivo experiments with cells such as fibroblasts. The clock was initially designed for precise age prediction of human fibroblasts and other skin cells, a feature in which previous clocks failed. Interestingly, this clock also showed good results with other samples, as sorted neurons, liver, or bone samples. It was notably used to estimate the epigenetic age acceleration (EAA) in Hutchinson-Gilford progeria syndrome, a very rare childhood disorder causing accelerated ageing [35, 36].

Horvath’s latest clock, GrimAge [37], dates from 2019. It is composed of seven DNA methylation-based estimators of plasma protein levels and one of smoking pack-years, a clinical quantification of tobacco exposure. Plasma cells are good age indicators; as a matter of fact, biological ageing is associated with changes of the plasma proteome [38]. GrimAge measures the EAA by comparing biological age and chronological age. The clock can thereby predict lifespan but also provide information on risks of age-related conditions such as coronary heart disease.

Other scientists have also devoted their time to the design of new epigenetic clocks. Levine et al. developed PhenoAge in 2018 [39], a highly accurate multi-tissue clock based on 513 CpG markers that predicts phenotypic age rather than chronological age. Phenotypic age derives from clinical biomarkers predictive of mortality such as creatinine concentration, mean red cell volume, or lymphocyte percent. From a cohort of people of the same age, PhenoAge can predict, among others, differences in the risk of all-cause mortality, cause-specific mortality and facial ageing.

The latest clock PedBE [40], developed by McEwen et al. in 2019, is very distinct from other clocks, since it is designed for paediatric populations. In young populations, DNA methylation is more dynamic and the biomarkers are different [41]. Given the targeted population, the test is non-invasive and is performed on buccal epithelial cells. Methylation is measured at 94 CpG sites. The clock can predict age with a median absolute error of 0.35 years. While McEwen et al. could have designed a clock for both paediatric and adult populations, they decided to focus only on paediatric populations to achieve higher accuracy. Comparing DNA methylation age and chronological age provides information on risks of paediatric diseases and on potential developmental defects. The clock was successfully used by its designers to study autism spectrum disorders (ASD) [40]. McEwen et al. observed a higher PedBE age for children with ASD compared to typically developing ones. ASD being associated with an increased body growth and an accelerated cortical development, the authors suggest that contrary to adults, an accelerated clock may be associated with development and not decline of biological functions. Moreover, gestational age was positively correlated with PedBE age acceleration, suggesting the implication of epigenetics in brain maturation. However, these studies are still at an early stage and need to be supplemented with results from larger cohorts. Nevertheless, this clock has an undeniable potential that should be harnessed to study the biological development of paediatric populations.

As two individuals of the same age do not necessarily have the same health, chronological age is a poor parameter to study ageing. Contrariwise, epigenetic age is a relevant variable as it integrates the impact of the environment. Thus, epigenetic clocks can not only provide information on the biological age but also on the ageing rate and on risks of age-related conditions. The clock should be carefully selected depending on the study, and results should always be confronted to another clock’s measure to ensure their accuracy. Building a wide range of precise epigenetic clocks, be they general, specific to a disease or to a type of population, will provide an extensive toolset to researchers to study ageing but also age-related diseases.

## Factors accelerating the biological age

**Fig. 2:**
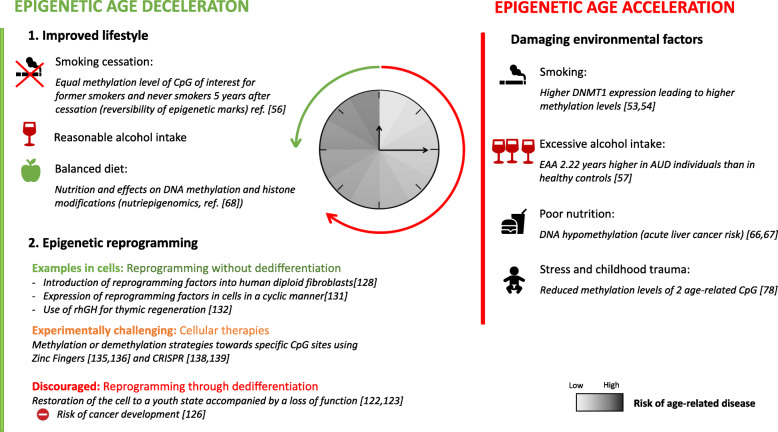
Examples of the effect of environmental factors and epigenetic reprogramming techniques to counteract ageing

Over the past two decades, numerous studies have been conducted on factors impacting epigenetic ageing. The effect of different external and internal stimuli on ageing may be difficult to interpret as the genome of each individual also plays a role in ageing. Therefore, cohorts of twins have raised an interest in the scientific community as even if they are genetically identical, and they exhibit an increasing number of phenotypic differences throughout their life [42]. A pioneering study in the field was the one of Fraga et al. [43], in which they studied eighty monozygotic twins with an age ranged from 3 to 74 years old. While looking at methylation and histone acetylation levels, Fraga et al. observed that the older the pair of twins was, the more distinct their epigenetic patterns were. This divergence was especially exacerbated when the twins were having very different lifestyles. This first epigenetic study on twins has been the key to show that twins are born with nearly identical epigenomes that start diverging as they age. It has opened the path to genome-wide association studies (GWAS) in monozygotic twins and Turner et al. [44] recently highlighted divergence in cohorts of twins aged 11, 17 and 23-24 years old. While older twins had left the parental home, younger pairs were still sharing the same household. Nevertheless, discordances of perception of various events, such as the television being always turned on, were observed between the two twins. The case co-twin studies thus provide new perspectives for investigating the impact of the natural and social environment on ageing, diseases, allergies, etc. [45–47]. Another example of twin research is the study conducted by the NASA on two male monozygotic twins, one sent in space for a year and one not. This work characterized the epigenetic modifications in the individual pre-flight versus inflight. Although the mean global methylation for this individual varied, the fluctuations were smaller than the ones observed for the twin who stayed on Earth. The twin who stayed on Earth during the same period underwent different epigenetic modifications [48]. In addition, telomere length increased during the flight period but shortened to the original length in the 48 h following return on Earth, maybe due to increased stress. As shown by this study, the differences in lifestyle such as diet, physical work and weightlessness for example induced these modifications. A better understanding of the impact of various factors traced by the epigenetic modifications can have a wide range of applications, from detection and treatment of age-related diseases to safe space trips. Herein, we chose some examples of environmental factor and report investigations on the influence of addictions such as nicotine, alcohol, nutrition, and stress on the epigenome (Fig. 2).

It is well known that smoking leads to increased risks of respiratory [49] and cardiovascular [50] diseases as well as different forms of cancer [51]. Cigarettes contain highly toxic compounds such as nicotine and provoke inflammation and genetic alterations in lung tissues among other pathogenic mechanisms [52]. In terms of epigenetic alterations, cigarette smoking induces changes in DNA, such as modified DNA methylation patterns [53]. Experimentally, DNMT1 expression was found to be significantly higher in lung tissues of smokers than of non-smokers [54] (Fig. 2) leading to an increase in methylation levels [55]. Other epigenetic marks such as histone hyperacetylation were also observed in the lungs of individuals with smoking-triggered chronic obstructive pulmonary disease [56] (COPD). Fortunately, as smoke-induced DNA methylation is reversible, comparison of methylation levels of CpG sites of interest between never smokers and former smokers showed little to no differences 5 years after cessation [57] (Fig. 2).

Influence of other addictions such as alcohol on DNA methylation patterns was also investigated (Fig. 2). Recent research works aimed at evaluating how alcohol use disorder (AUD) severity is correlated to epigenetic age acceleration [30]. Epigenetic age of the study’s participants was determined by input of clinical measures in various epigenetic clocks. EAA was then calculated as the difference between the epigenetic age and the chronological age. For example, Levine’s DNAm PhenoAge clock [58] indicated a mean EAA value of AUD patients that was 2.22 years higher than the one of healthy controls [30]. As it is difficult to isolate the influence of a specific environmental factor, additional covariates such as smoking status and body mass index were considered [59]. After adjustment, individuals with AUD demonstrated a lower but still relevant EAA compared to healthy controls.

As alcohol and cigarette consumption are intertwined, both influences were studied at the same time looking at the methylation status of two distinct specific CpG sites by Beach et al. [60]. Surprisingly, the results using the first epigenetic clock designed by Hannum [32] gave two different trends. Although DNA methylation increases at specific sites with age, smoking and alcohol consumption displayed divergent effects on biological ageing. At CpGs for which DNA methylation increases with age, alcohol use prevented this methylation, while smoking had little effect. At the CpG sites where methylation decreases with age, both alcohol consumption and cigarette smoking were promoting methylation. As a result, despite their link, alcohol and cigarettes consumption have a different effect on biological ageing. As previously stated, this clock is only focusing on 71 CpG, which is not representative enough. Newer epigenetic clocks, perhaps solely dedicated to the study of smoking and AUD, need to be used to provide clearer correlations.

Nutrition also plays an important modulatory role in ageing and age-related diseases [61]. Nutrients have two different mechanisms of action involved in methylation reactions [62]. Folate, for example, is a precursor of methionine [63] from which SAM is synthetized [64], while serine promotes de novo synthesis of ATP, which activates the transformation of methionine into SAM [65]. More globally, both nutrients regulate levels of the SAM cofactor and of the side product of the methylation reaction, *S*-adenosyl-l-homocysteine, which inhibits methylation. The SAM cofactor is essential to DNA and histone methylation. Nutrient deficiency can induce DNA hypomethylation with severe consequences such as cancer development as liver cancer [66, 67]. Complementation with polyphenols as green tea catechins (GTCs) have been reported to have various effects on the epigenome [68] (Fig. 2). In vitro GTCs were able to lower the DNA methylation level of promoters of genes involved in DNA reparation, for example, restoring their initial function [69]. However, a following in vivo study on mouse showed no inhibition of DNA methylation in normal and cancerous murine prostate, gut and liver [70]. Other polyphenols found in black raspberries were proved to prevent and inhibit the progression of colon cancer in rodents [71]. These results were confirmed in humans by a small clinical study conducted on patients suffering from colon cancer. As a consequence of daily black raspberries intake, DNMT1 levels were reduced, and DNA demethylation was observed on promoter of genes involved in proliferation [72]. Lastly, some isoflavones found in soya, like genistein, were reported to be cancer-preventive compounds as diet rich in soya have shown to induce a lower risk of hormone-dependent cancer [73, 74]. Although epigenetic modifications linked to nutrition have been explored in the past two decades, their mechanisms and their impact on cancer prevention are not always well established. Further experimental studies are required in order to achieve personalized nutrition-based therapies to delay age-related diseases [75].

Lastly, stress is said to play an important role in epigenetic modifications whether it comes from depression [76] or childhood trauma [77], for example (Fig. 2). This effect was investigated by looking at FKBP5, a protein involved in stress physiology, in three different cohorts gathering up to 3000 people [78]. Two age-related CpG sites were found to have reduced levels of methylation in all cohorts. In addition, these two sites were close to each other and upstream of the transcription region of FKBP5. Experiments were performed in vitro applying stress hormones to human fibroblasts, and an inverse relationship between ageing and FKBP5 methylation was confirmed. As depression is strongly associated with early life trauma [77], the impact of childhood trauma on FKBP5 methylation modulation was studied [78]. It was found to decrease methylation at the two previously mentioned CpG sites as well. An additional study calculated the epigenetic age using all the methylation sites in blood and post-mortem brain samples [79]. Patients with major depression had a significantly higher epigenetic ageing than healthy controls. This difference was especially higher in individuals who experienced childhood trauma. Overall, stress is found to be acting along ageing to reduce DNA methylation at specific sites, such as the two CpG sites found upstream of FKBP5. This triggers an enhanced FKBP5 response in immune cells, which later promotes dysregulation of NF-κB, a protein complex implicated in the regulation of the immune response to infection.

## Epigenetics and age-related diseases

Geroscience states that age is a major risk factor of age-related diseases [80], suggesting that age-related pathologies are an inevitable outcome of ageing. Changes in the epigenome that accompany ageing promote age-related conditions, such as cancer, Alzheimer’s disease and chronic inflammation [81]. Although epigenetic clocks are based on DNA methylation levels, methylation is not the only epigenetic mark involved in ageing. Histone methylation and acetylation changes have also been observed during ageing [82, 83]. Commonalities and divergences between ageing and the three discussed age-related conditions (chronic inflammation, cancer, and Alzheimer’s disease) are documented at the end of the section in Table 1.

**Table 1:** Commonalities and divergences between ageing and age-related conditions (chronic inflammation, cancer and Alzheimer’s disease)

	Chronic inflammation		Cancer		Alzheimer’s disease	
**Commonalities with ageing**^a)^	**Epigenotype**	**Genotypic and phenotypic outcome**	**Epigenotype**	**Genotypic and phenotypic outcome**	**Epigenotype**	**Genotypic and phenotypic outcome**
	Chromatin remodelling and di- and tri-methylation of histone H3K4 [82, 87]^b)^	Increased level of inflammatory cytokines [86]	Common methylation patterns (epigenetic drift)	Cell vulnerable to mutations [96, 97]	Redistribution of H4K16ac [110]^b)^	Changes in the expression of nearby genes
	Age-related DNA methylation changes on key genes [142] (e.g. hypomethylation of the tumour necrosis factor (TNF) promoter [88])	Gene deregulation promoting inflammation	Global DNA hypomethylation and specific promoter hypermethylation In cancer: hyper-methylation of tumour suppressor gene promoters and hypomethylation of repetitive sequences	Silencing of tumour suppressor genes [98] and genome instability [99, 100]	Accumulation of 5hmC, 5fC and 5caC [111, 113, 114]	Dysregulation of mechanisms involved in brain development and function
	Overexpression of histone acetyl-transferase P300 [83, 89]	Premature senescence and inflammation	Global methylation level decrease [102], 8-oxo-deoxyguanosine level increase [84]	Low methylation and high 8-oxo-deoxy-guanosine levels are associated with an increased glioma malignancy grade [103]		
**Divergences with ageing**			Deregulation of epigenetic ageing rate associated with cancer malignancy [96] (e. g. lower epigenetic age in gliomas is associated with poor survival [94])		- Overall increase of H4K16ac upon ageing and global loss in AD subjects [110] - 27 signatures of AD are age-independent (19 for 5mC, 5 for 5hmC, 3 for 5fC/caC) [111]	

^a^In the disease the common trait is exaggerated

^b^H3K4 = lysine 4 on histone 3, H4K16ac = acetylation of lysine 16 on histone 4

Ageing is also characterized by DNA damage accumulation [84], leading among other effects to telomere attrition and inevitably to senescence [85]. Senescence is a normal outcome of ageing: it is a primitive defence mechanism that translates into a cell-cycle arrest to avoid proliferation of damaged cells. Abnormal accumulation of senescent cells promotes inflammation and cancer via senescence-associated secretory phenotype (SASP). An increased level of inflammatory cytokines upon ageing has also been observed [86]. Mezayen et al. have found that chromatin remodelling characterized by di- and tri-methylation of histone H3K4 upon ageing leads to overexpression of *P19*, a subunit of the inflammatory cytokine interleukin 23 (IL-23), which in turn leads to the expansion of T helper-17 cells responsible for proinflammatory responses [87]. Moreover, Gowers et al. [88] showed age-related DNA hypomethylation on the tumour necrosis factor (TNF) promoter, thereby upregulating its expression and promoting inflammation. Sen et al. [89] identified histone acetyltransferase (HAT) P300 as a key actor of pro-senescence changes by high-throughput screening. They found that depletion of HAT p300 delays senescence, while its overexpression induces premature senescence and thereby inflammation. The effect of HAT p300 expression on senescence makes it a potential therapeutic target for rejuvenation. Chronic inflammation promotes numerous age-related pathologies, such as cance r [90] and neurodegenerative diseases [91, 92], hence the need to prevent it. However, tackling chronic inflammation without altering normal senescence processes represents a big challenge. Although senescence promotes cell proliferation and inflammation via SASP, it is also the primitive defence mechanism against cancer and is essential for the recruitment of immune cells. Choosing a therapeutic target involved in senescence mechanisms is a tricky path.

Numerous studies have been dedicated to cancer [80, 93–96]. Some have shown common DNA methylation patterns between aged cells and cancer cells, suggesting that the epigenetic drift is making cells vulnerable to mutations and therefore more prone to cancers [96, 97]. A global DNA hypomethylation as well as a local DNA hypermethylation of CpG islands is also observed in cancer cells. Hypermethylation of CpG islands on tumour suppressor gene promoters leads to the silencing of these key genes [98]. Hypomethylation of repetitive sequences activates their transcription and causes genome instability [99, 100]. An epigenetic clock, epiTOC [101], was specifically designed by Yang et al. to predict cancer risk by estimation of the number of stem cell division in a tissue. This mitotic clock is accelerated in cancerous and precancerous cells but also in cells exposed to carcinogens. Epigenetic ageing was investigated in glioma by Liao et al*.* [94] using epiTOC [101] and Horvath’s first clock [28, 29]. Horvath’s clock was chosen to estimate epigenetic age in tumours for its precision despite heterogenicity of the tumour. EpiTOC completes the result by providing information on cell division. The study revealed that each glioma subtype has its own epigenetic imprint. Counterintuitively, lower epigenetic age is associated with poor survival. The authors suggest that there might be a biological mechanism in gliomas running counter to epigenetic ageing yielding an increased aggressivity and treatment resistance. No relation between DNA methylation patterns and tumour recurrence was found. Global DNA methylation can also be used as a relevant biomarker for ageing and age-related diseases, as it has been shown that it decreases upon ageing [102] and in cancer. Global DNA methylation and 8-oxo-deoxyguanosine—a specific marker of oxidative-stress derived DNA damage—levels were used as biomarkers by Barciszewska et al. to study gliomas malignancy [103]. The authors pointed out an inversely proportional relationship between global DNA methylation and 8-oxo-deoxyguanosine levels. The authors explain this inversely relationship by the fact that oxidation of the guanine at methylated CpG sites induces cytosine demethylation by recruiting the ten-eleven translocation (TET) enzymes. Importantly, low DNA methylation and high 8-oxo-deoxyguanosine levels were associated with an increased glioma malignancy grade.

Indeed, in cancer, a global DNA hypomethylation is observed together with an hypermethylation of specific gene promoters [104]. For example, certain diagnostic kits are based on the detection of the hypermethylation of defined genes for each cancer type (as EpiproLung®, EpiproColon®, ColoGuard®). Beyond cancer, DNA methylation can be used for diagnostic and prognostic of other diseases. Low levels of DNA methylation are associated with other age-related conditions such as hypertension. Similarly to Barciszewska et al., Smolarek et al. [105] identified an opposite trend between the hypertension’s severity and the DNA methylation level, independently from other clinical and biochemical factors. In this case, global DNA methylation was used as a gauge to evaluate its severity and monitor its progression. Another interesting use of DNA methylation is in the response to treatment. In glioblastoma, the hypermethylation of O^6^-methylguanine DNA methyltransferase (MGMT) is determinant for administrating temozolomide. There are also studies assessing whether the restoration of normal methylation level in patients is indicative of response to treatment and/or recovery [106, 107].

Another age-related pathology of public health concern is Alzheimer’s disease (AD). Ageing is the major risk of AD [108], a disease characterized by the accumulation of intercellular β-amyloid plaques and intracellular neurofibrillary tangles [109]. Nativio et al. have studied epigenetic changes associated with AD and ageing by comparing brain samples from young and old cohorts as well as AD subjects [110]. The study revealed a redistribution of acetylated H4K16 (H4K16ac) in AD. An overall increase of this epigenetic mark is observed upon ageing, whereas a global loss is observed in AD subjects. H4K16ac changes in AD can be divided into 3 groups according to their location: age-regulated (common gain of H4K16ac for AD and old subjects, but not young subjects), age-dysregulated (loss of the mark for AD and young subjects but gain for old subjects) and disease-specific (loss only for AD subjects). Location of the mark influences the expression of adjacent genes. Recently, Fetahu et al. discovered an epigenetic signature in DNA methylation patterns in neurons peculiar to AD [111]. In addition, to methylated cytosine (5mC), they also focused on 5-hydroxymethyl-cytosine (5hmC), 5-formyl-cytosine (5fC) and 5-carboxylcytosine (5caC). These oxidized derivatives of 5mC are intermediate products in the demethylation reaction catalysed by the TET enzymes [112]. Decarboxylation of 5caC yields, through DNA repair, the unmethylated cytosine. Accumulation of these oxidized products is a normal biological phenomenon during brain development and function [113, 114], suggesting that AD emergence and progression is due to a dysregulation of these mechanisms. Fetahu et al. [111] conducted a deep study. Results obtained on cell cultures of normal cell lines (wild type), early-onset familial AD cell lines (PSEN1 and PSEN2) and late-onset familial AD cell line (APOE4) were validated on post-mortem brain tissues of healthy donors and AD patients. Twenty-seven age-independent signatures of AD—19 for 5mC, 5 for 5hmC and 3 for 5fC/caC—were identified. Genes carrying the signature were either involved in neurodevelopment and neuronal transcription factors, in critical cellular processes, in RNA associated proteins or in cell signalling. Further research on these 27 signatures led to the identification of 39 CpG sites that are AD specific—27 related to 5mC, 4 to 5hmC and 8 to 5fC/caC—that differ in early-onset, late-onset, familial and sporadic AD models. This extensive study aimed at providing tools to early-diagnostic and quantify the risk of AD.

A better understanding of the mechanisms behind age-related diseases is essential for early diagnostic and for the design of an efficient therapy. Epigenetic clocks are real assets to understand these mechanisms and to determine the risk of an age-related condition.

## Reversing the time

The reset of the ageing clock is a natural biological process that occurs at every fertilization event. In humans, the ageing clock is set back to zero by erasing the marks of ageing in the two zygotes leading to a young single cell: the embryo [115]. Studies of the fertilization event showed that the donor nucleus is reprogrammed by the oocyte cytoplasm [116]. As in a new fertilization event, the characteristics of ageing are erased in the epigenome during the epigenetic reprogramming (Fig. 2) and set back to the stage of youth cells [117]. Being able to reset the ageing clock offers numerous applications in therapies but also in cloning. In 1996, Dolly was the first mammal clone generated from adult somatic cells that turned into a fertile adult sheep without premature ageing signs [118]. Although this first sheep died earlier than expected due to osteoarthritis [119], additional studies on older clones from the same cell line as Dolly and other cloned sheep showed little to mild osteoarthritis and required no treatment unlike Dolly [120]. Therefore, globally, these cloning events did neither affect the healthspan of this group of sheep nor induce a faster ageing.

Rejuvenation consists in the restoration of the cell to a youth state accompanied by a loss of function: the cell is dedifferentiated [121] (Fig. 2). Takahashi et al. showed that differentiated adult mouse fibroblasts can be reprogrammed into induced pluripotent stem cells by the transfer of only four master genes: oct4, sox2, klf4 and c-myc (OSKM genes) [122]. The cells can later be differentiated again into the desired cell type using differentiation factors. For fibroblasts, it has been observed that rejuvenated cells have similar characteristics as young natural fibroblasts cells, such as low levels of oxidative stress [123]. However, this method needs to be improved to increase the percentage of cells reaching pluripotency [124]. In addition, dedifferentiation increases the risk of cancer development by forming teratomas [125]. Consequently, many research studies have been focusing on resetting the ageing clock without a dedifferentiation stage for therapeutic applications like transplantation [126]. One of the methods consists in introducing reprogramming factors into human diploid fibroblasts [127]. Horvath’s clock [28, 29] was used to monitor the decrease in epigenetic age with an initial epigenetic age around 65 years. The epigenetic age of the cells decreased at a rate of 3.8 years per day, and no pluripotency gene was expressed before the decrease in epigenetic age. There was no expression of genes specific to the fibroblasts 15 days after the epigenetic age reached zero [128]. This shows that the loss of the fibroblast identity was achieved well after the completion of cell reprogramming. It is thus possible to reprogram cells without going through dedifferentiation. Direct observation of these epigenetic changes was possible as well thanks to heterochromatin protein 1 beta (HP1β) [129]. A study in mice established that the mobility of this protein depends on the differentiation state of the cell. HP1β is more dynamic in undifferentiated cells than in senescent ones and can keep track of the progressive loss of the cell’s function. Another rejuvenation method consists in expressing reprogramming factors in cells in a cyclic manner [130]. No loss of differentiation markers and expression of pluripotency markers was observed, indicating that age reprogramming in vivo can be achieved in the absence of developmental reprogramming.

Reprogramming of aged cells to a youthful state carries risk of tumour-development. Avoiding the loss of cell differentiation may be a more secure alternative (Fig. 2). In 2015–2017, the first human clinical trial investigating the possibility to reverse ageing was designed using recombinant human growth hormone (rhGH) for thymic regeneration, which is crucial for a sufficient immune cell reservoir [131]. Fahy et al. followed rejuvenation using Horvarth’s pan tissue clock [28, 29] and confirmed the result with three other clocks: Hannum’s clock [26], PhenoAge [33] and GrimAge [31]. All four epigenetic clocks measured the same trend and showed decrease of the epigenetic age of about 2.5 years after 12 months of treatment. Although this deceleration tended to reverse 6 months after cessation of the treatment, there was still a notable 1.5 years decrease in epigenetic age. Even if this age regression is within the range of the reported error of the various epigenetic clocks, such as 3.6 years for Horvath’s first clock [28, 29], the observed age regression is supported by four clocks built on different models and based on different CpG sites, comforting an age-regression. Moreover, it was accompanied by significative changes in a variety of age-related immunological parameters. A notable increase in the thymic fat-free fraction (TFFF) was observed in most patients indicating that the thymic involution process was reversed. In addition, the lymphocyte-to-monocyte ratio (LMR) increased as a result of the CD38^+^ monocyte percentage decline. Higher LMRs have been linked with a reduced mortality for numerous diseases such as stroke [132]. These data thus support a thymic rejuvenation. However, this work must be considered carefully as the clinical trial was only based on nine individuals.

Another approach to reverse epigenetic time consists in inverting the DNA methylation process occurring with ageing (Fig. 2). As mentioned previously, as the individual gets older, the genome is globally hypomethylated, while the CpG sites of particular gene promoters are hypermethylated [25, 26]. Restoring the balance of the DNA methylation level could re-establish the genome stability. With recent development of precise biological tools, it becomes possible to modify the DNA methylation status at a specific locus. Gene-targeting strategies such as zinc finger proteins (ZFPs) have been used to target specific locations in the genome [133]. Experimentally, ZFPs associated to DNA methyltransferase catalytic domains were successful in methylating more than 10 out of the 12 CpG sites in the gene promoter of the vascular endothelial cell growth factor A [134] and the promoter of the oncogene SOX2 [135]. However, as ZFPs are designed for a specific sequence [136] and are laborious to setup, it is hardly conceivable to target all age-related CpGs. Even though only 2% of the total CpG sites show age-related methylation changes, it still represents more than 500,000 CpG sites out of the 28 million CpG sites of the whole genome [17]. Thus, this method could be considered if only very few key game-changing CpGs are identified. Further, studies are needed. Other epigenetic rewriting strategies use CRISPR/dCas9 constructs [137, 138]. This method is more flexible since it can be easily redirected toward a new target by changing the RNA guide [139]. Moreover, Stepper et al. [140] observed a spread of the methylation to an entire CpG island with a dCas9 methyltransferase construct directed towards a single CpG site. Knowing that CpG islands are mainly located on gene promoters, the spreading could be harnessed to target specific gene promoters and thereby modulate the expression of the corresponding gene.

Rejuvenation techniques are crucial to erase the effects of ageing and damaging environmental factors (Fig. 2). Reversing the time is of prime importance in the prevention of age-related diseases. Novel methods have allowed a safer epigenetic reprogramming, and studies on genome-engineering techniques are encouraging. Nevertheless, further studies are needed to evaluate the durability of the epigenetic modifications, as well as the application of CRISPR/dCas9 system to ageing in humans, and the safety.

## Conclusions

Due to the ageing of the population, there is a rising concern to assure the well-being of older individuals by expanding their healthspan. Adopting the epigenetic perspective of ageing is promising. Epigenetic age is a key parameter to assess the health status of an individual and to monitor ageing. Epigenetic clocks were used to explore the influence of environmental factors on the DNA methylome and thereby ageing (Fig. 1). Studies indicate high alcohol and cigarette consumption, poor diet and stress as damaging environmental factors and reasonable alcohol intake, and no smoking and balanced diet as beneficial factors (Fig. 2). Epigenetic clocks can also be used to monitor and anticipate age-related diseases, thereby allowing treatment at a very early stage. Reversibility of epigenetic modifications makes them promising therapeutic targets for age-related disease treatments as well as for rejuvenation strategies. In the last few years, studies have been published describing age-reversing techniques. Initial rejuvenation methods involving a loss of cell differentiation were replaced by epigenetic reprogramming to avoid any possible tumour development [125]. The high potential of epigenetic reprogramming has been highlighted in a recent clinical trial showing a mean 2.5 years regression in epigenetic ageing after 12 months of treatment [131]. However, the low number of patients of the study does not allow to conclude on the efficiency of the treatment. Hopefully, studies on larger cohorts will allow to conclude whether the discussed rejuvenation methods are efficient. Lastly, genome-editing methods such as the CRISPR/dCas9 construct have been proved to be efficient at methylating large regions beyond the initial targeted CpG site [140].

The field is at its beginning, and there are several challenges. While some epigenetic mechanisms involved in age-related pathologies have been identified, the ones behind ageing remain poorly known (Table 1). In order to prevent ageing, it is of particular importance to determine how epigenetic writers and erasers are directed towards specific sites to implement or remove a mark. DNA and histone methylation patterns associated with ageing have been extensively studied, perhaps neglecting the role of phosphorylation, histone variants, remodelling complexes or non-coding RNAs.

Moreover, it is crucial to identify the factors responsible for the clock acceleration or deceleration. While trends have been identified, no firm conclusions can be drawn from these studies. To date, studies of large cohorts of monozygotic twins remain the most reliable way to study the effect of environmental factors on the epigenome as it removes the genomic variable. However, the impact of each environmental factor on epigenetic age is hard to determine, epigenetic age being a function of many variables and happenstance. Isolating the effect of a single factor on ageing is a great challenge. Sequencing the epigenome of each individual at regular intervals throughout their lives while keeping records of their lifestyle and environment changes might allow to establish more accurate links between epigenetic modification and an environmental factor.

Finally, although genome-editing methods have delivered promising results in vivo for epigenetic reprogramming, some major concerns remain un-addressed. Clinical use of these systems is still underexplored [141], and the safety in time needs to be addressed. Moreover, as DNA methylation of CpG sites is reversible, it is crucial to obtain more data on the long-time efficiency of the chemical modifications brought by genome-editing methods.

## Data Availability

Not applicable

## Abbreviations

CpG: Deoxycytidine-phosphate-deoxyguanosine dinucleotide
DNMT: DNA methyltransferase
SAM: *S*-adenosyl-l-methionine
EAA: Epigenetic age acceleration
ASD: Autism spectrum disorder
GWAS: Genome-wide association studies
COPD: Chronic obstructive pulmonary disease
AUD: Alcohol use disorder
GTC: Green tea catechins
SASP: Senescence-associated secretory phenotype
IL-23: Interleukin 23
TNF: Tumour necrosis factor
HAT: Histone acetyltransferases
MGMT: O^6^-methylguanine DNA methyltransferase
AD: Alzheimer’s diseases
5mC: Methylated cytosine
5hmC: 5-hydroxymethyl-cytosine
5fC: 5-formyl-cytosine
5caC: 5-carboxylcytosine
TET: Ten-eleven translocation enzyme
HP1β: Heterochromatin protein 1 beta
rhGH: Recombinant human growth hormone
TFFF: Thymic fat-free fraction
LMR: Lymphocyte-to-monocyte ratio
ZFP: Zinc finger protein
CRISPR/dCas9: Clustered, regularly interspaced short palindromic repeats/dead native Cas9 nuclease
